# Antioxidant and neuromodulatory effect of gallic acid prevents anxiety-like symptoms of omeprazole

**DOI:** 10.1039/d5ra09924b

**Published:** 2026-02-17

**Authors:** Noreen Samad, Natasha Manzoor, Shehreen Saleem, Saima Khaliq, Umer Ejaz, Mahwish Arshad, Sadaf Ahmad, Bakar Bin Khatab Abbasi, Ali Irfan, Yousef A. Bin Jardan

**Affiliations:** a Department of Biochemistry, Faculty of Science, Bahauddin Zakariya University 60800-Multan Pakistan noreen.samad@bzu.edu.pk natashamanzoor.nm@gmail.com shehreen_saleem@yahoo.com umarejaz5757@gmail.com; b Department of Biochemistry, Federal Urdu University of Art, Science and Technology Karachi Pakistan saima.khaliq@fuuast.edu.pk; c Roy & Diana Vagelos Laboratories, Department of Chemistry, University of Pennsylvania Philadelphia Pennsylvania 19104-6323 USA maarshad@sas.upenn.edu; d Department of Medicine, Perelman School of Medicine, University of Pennsylvania Philadelphia 19104 USA; e Department of Physics, Chemistry and Pharmacy, University of Southern Denmark Campusvej 55 5230 Odense Denmark sadaf@sdu.dk; f Department of Chemistry and Chemical Biology, Northeastern University Boston MA 02115 USA abbasi.ba@northeastern.edu; g The Green Institute of Chemical, Biomedical and Environmental Sciences (GICBES) Lahore-54000 Pakistan draliirfan.ceo@gicbes.com raialiirfan@gmail.com; h Department of Pharmaceutics, College of Pharmacy, King Saud University Riyadh 11451 Saudi Arabia ybinjardan@ksu.edu.sa

## Abstract

This research focuses on the protective impacts of gallic acid (GAL) on OMP-instigated anxiety. In this experiment, 48 male Sprague-Dawley rats were grouped into 6 (*n* = 8): (I) VEH + VEH (1 mL kg^−1^), (II) VEH + GAL [low dose; (50 mg kg^−1^)] (III) VEH + GAL [high dose; (100 mg kg^−1^)] (IV) VEH + OMP (20 mg kg^−1^), (V) GAL (low dose) + OMP V) GAL (high dose) + OMP. The animals were administered their corresponding drugs intraperitoneally (IP) for four weeks, once daily. Subsequently, during the treatment period, behavioral tests were performed, including open field activity (OFA) and home cage activity (HCA) to assess locomotion and elevated plus maze (EPM), and light-dark activity (LDA) for anxious-like symptoms, respectively. Following behavioral assessments, the rats were decapitated, and their hippocampus was removed. The hippocampus was utilized for biochemical, neurochemical, and histopathological examinations. The results revealed that OMP produced hypolocomotion, anxiety-like behavior, elevated oxidative-stress biomarkers and inflammatory-cytokines, and reduced the activity of antioxidant enzymes and serotonin metabolism. Administration of GAL (both doses) increased (*p* < 0.05) locomotor activity, reduced (*p* < 0.05) anxiety, decreased (*p* < 0.05) oxido-neuroinflammation, and normalized (*p* < 0.05) 5-HT metabolism and histopathological alteration, followed by OMP. 5-HT1A receptor analysis exhibited OMP-administration sensitized relative expression of 5-HT1A receptors, while GAL at both doses (*p* < 0.05) decreased 5-HT1A receptor expression in VEH and OMP-administered rats. It is proposed that GAL, as an antioxidant and neuromodulator, can be used for reducing OMP-induced hypolocomotion, anxiety, and associated neurological problems.

## Abstract 2

This research focuses on the protective impacts of gallic acid (GAL) on OMP-instigated anxiety.

## Graphical abstract

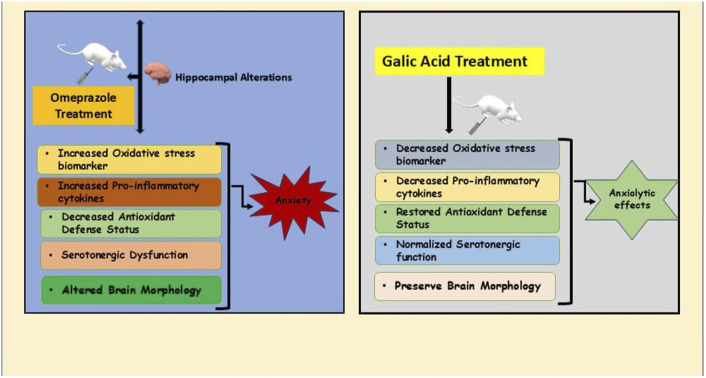

## 1. Introduction

Omeprazole (OMP) is widely prescribed globally as a prominent proton pump inhibitor (PPI) for addressing gastroesophageal reflux and hyperacidity.^1^ It is a part of the benzimidazole class, and in the stomach, it blocks the H^+^-K^+^ ATPase proton pump.^2^ About 40–50% of OMP is orally bioavailable in humans, and the half-life in the plasma is < 1-h, while it is completely removed in ∼3–4 h.^3^ The oral LD_50_ of OMP in mice is very high (>4 g kg^−1^). At lower repeated doses (125 µmol per kg per day ≈50 mg kg^−1^) of OMP, rodents sometimes show mild anemia and, after long durations, gastric ECL hyperplasia.^4^ Chronic OMP (≈20–30 mg kg^−1^) can inconsistently enhance weight gain under high-caloric feeding by lowering metabolic rate.^5^ The acute OMP effect on weight is less studied; however, very high acute doses (40–150 mg kg^−1^ i.p.) slow gastrointestinal transit and might affect feeding indirectly.^6^ Repeated OMP administration upsets glucose homeostasis in BALB/c mice given 20 mg kg^−1^ OMP daily for 30 weeks, fasting blood glucose and HOMA-IR rose significantly compared to controls.^7^ Studies have shown that OMP impaired redox status balance and enhanced oxidative-stress.^1,2^ In previous animal studies low and high doses (10 and 20 mg kg^−1^; b.w) of OMP were administered intraperitoneally, but only 20 mg kg^−1^ injected animals induced anxiety-like behavior by affecting 5-HT receptors in the hippocampus, altering 5-HT metabolism in experimental animals.^8,9^

Anxiety is an adaptive reaction to the possible occurrence of danger.^10^ It is a prevalent mental health issue with a regional disparity in occurrence worldwide (*i.e.*, from 5.3% to 10.4%^11^ and a collective lifetime occurrence of ∼12.9%.^12^ This disorder manifests as excessive worry, fear, and apprehension about future events, often leading to debilitating emotional distress and physical symptoms.^13^ The origin of anxiety at the neurobiological levels can be considered commotion in the basic mechanisms controlled by the Hypothalamic-Pituitary-Adrenal (HPA) axis. Therefore, anxiety involves various brain structures that regulate the HPA axis, *i.e.*, the hypothalamus, amygdala, periaqueductal gray, and hippocampus.^14,15^ The hippocampus, is intricately linked to anxiety regulation, acting as a vital hub for memory consolidation and emotional processing, due to perturbations such as oxidative stress, inflammation, and disruptions in serotonin and other neurotransmitter levels.^16–18^

In the pathogenesis of anxiety, several neurotransmitters have been implicated. Earlier studies highlighted the role of monoamines, *i.e.*, serotonin (5-Hydroxy-Tryptamine, 5-HT), in anxiety.^19^ 5-HT, assists as a neurochemical, exerting its effects at central and peripheral levels.^20^ About 14 distinct types of serotonin receptors are present in the brain. Among these, 5-HT-1A is a notable subtype of the 5-HT receptor.^21^ Behavioral deficits, *i.e.*, depression and anxiety, are influenced by 5-HT in behavioral and emotional processes.^22^ Both presynaptic and postsynaptic receptors are present in serotonergic neurons. It is believed that the 5-HT1A somatodendritic receptors inhibit neuronal firing and play a major role in controlling serotonergic neurotransmission, whereas postsynaptic 5-HT-1A receptors also impact the proclamation of other neurochemicals. The abnormal neurotransmission and its receptor responsiveness cause anxiety.^23^

GAL is a tri-hydroxybenzoic acid and a polyphenol found in various fruits, vegetables, and medicinal plants.^24^ GAL and its dimer ellagic acid, digallic acid, and hexahydroxyidiphenic acid are considered naturally found phenolic lactones in the form of hydrolyzable tannins called ellagitannins.^25,26^ It has excellent anti-inflammatory and antioxidant effects^27^ by obstructing the instigation of TGF-β/NFKappa-β/MAPKs.^28^ GA pretreatment (10–50 mg kg^−1^) in ethanol-induced gastric ulcers lowered pro-inflammatory cytokines and lipid peroxides, while boosting endogenous antioxidants and protective mediators *via* the Nrf2/HO-1 pathway. Thus, GA alone strongly activates antioxidant/anti-inflammatory defenses in the gastric mucosa.^29^ A GA-rich fruit extract (Phyllanthus emblica) was tested with OMP in NSAID-induced gastric ulceration. The combination of OMP and GA-enriched extract accelerated ulcer healing more than OMP alone and reversed NSAID-induced inflammation and increased pro-healing factors (Chatterjee *et al.*, 2012). GAL also reduces various cancers, microbial activity, diabetes, obesity, *etc.*.^30–34^ It is also a neuroprotector of β-amyloid peptide (Aβ) in an *in vitro* study^35^ and has been used in the food, cosmetics, and pharmaceutical industries.^36^ Studies have been shown that GAL has the potential to prevent diet-induced weight gain. In a study, C57BL/6J mice fed a high-fat diet and treated with GA (100 mg per kg per day) gained less weight than high fat diet.^37^ On the other hand, very low dose (20 mg kg^−1^) of GAL did not improve weight or metabolic parameters.^38^ In diet-induced obese mice, GAL treatment (dose ∼100–200 mg kg^−1^) restored normoglycemia and insulin sensitivity without changing food intake.^39^ High-fat diet models demonstrated that GA lowers serum triglycerides, total and LDL cholesterol, and blood glucose.^37^ Earlier studies showed that GAL has anxiolytic, antidepressant, and cognitive-improving effects.^40^ In mammals, GAL is non-toxic at pharmacologically approved doses.^41^ GA doses required for metabolic effects (∼50–200 mg kg^−1^) are well below toxic levels; high-dose GA is essentially non-toxic in mice.^42^

Based on the anxiolytic and antioxidant effects of GAL in a previous report,^40^ the current work aimed to assess the outcome of numerous doses of GAL on OMP-instigated anxiety-like behavior, oxidative stress, neuroinflammation, impaired serotonergic mechanism, and hippocampal morphology in rats.

## 2. Materials and methods

### 2.1. Animals

48 Sprague Dawley rats (*n* = 8/group, adult males, weighing 170–190 gm; 7–8 weeks old) were randomly selected for this study. All the rats were housed individually (to avoid the effect of social interaction) and provided permitted admittance to food pellets and drinking water. A controlled environment was sustained at 26 ± 2 °C and 12 hours of day and night cycles. Before the experiments, animals were exposed to 7 7-day habituation period and to behavioral processes to abolish the psychological complaint of the situation to lessen originality and management stress. An approval (D-1893/2021/Biochem: Dated: April 11, 2021) was taken from the Departmental Bioethical Committee to conduct all experimental methods. The rats were ethically treated rendering to the procedures of “The Guide for the Care and Use of Laboratory Animals (Eighth Edition, 2011). It is declared that the present work is described under ARRIVE guidelines.

### 2.2. Chemicals

Gallic acid (GAL: Cat#7384), Omeprazole, Dithio-bis-nitrobenzoic acid (DTNB; Cat# Cat#D8130), Hydroxylamine-Hydrochloride (HONH_2;_ 159417), Potassium-Dichromate (Cat#P5271), Acetic-Acid, Sodium-Bicarbonate (Na_2_CO_3_), Disodium Hydrogen Phosphate, glutathione reduced (Cat#G4251), Sodium-Azide (Cat#S8032), Nitro-blue Tetrazolium (NBT; Cat#298839), and Trichloroacetic acid (TCA; Cat#T5500), IL-6 (Cat#ab234570; range 125–8000 pg mL^−1^) and TNF-α (Cat#ab100784; 82.3–20000 pg mL^−1^) *etc.*

### 2.3. Experimental schedule

In the treatment schedule, forty-eight rats were separated into Six groups (each comprising eight animals): (I) vehicle (VEH)+VEH; 1 mL kg^−1^ (II) VEH + GAL [50 mg kg^−1^ (ref. 40)], (III) VEH + GAL [100 mg kg^−1^ (ref. 40)] (IV) OMP [20 mg kg^−1^ (ref. 8)], (V) GAL [50 mg kg^−1^ ] + OMP [20 mg kg^−1^ ] (VI) GAL [100 mg kg^−1^ ] + OMP [20 mg kg^−1^ ]. The control group received distilled water as VEH, while the other groups received GAL and OMP. The dose selection of OMP and GAL based on reported work as indicated earlier, that OMP at this selected dose produced anxiety-like behavior,^8^ whereas, GAL at both doses^40^ produced anxiolytic effects in rats. The dose of OMP used in the study was equivalent to that used in the clinical setting.^43,44^ GAL and OMP (log *P* value = 1.68) were solubilized in distilled water and administered *via* intraperitoneal (IP) injections (*via* separate syringes) every day (8:30–9:30 am), the time interval between two injections was 2–3 minutes) for 28 days. On day 29 (from 8:00 am to 4:00 pm) open field activity (OFA) and home cage activity (HCA) were performed to assess locomotor activity in novel and familiar environments.^45^ Light dark activity (LDA) and elevated plus maze (EPM) activity were conducted on day 30 (from 8:00 am to 4:00 pm) for assessment of anxiety^40^ as reported previously. Simultaneously, animals received their respective treatment after the behavioral tests on their respective day. After the behavioral analysis, the animals were decapitated on day 31 (1-h after the drug administration). Subsequently, brain samples were extracted and promptly immersed in cold saline. The brain samples were then sited in a cutter with the adaxial side reported earlier.^46^ A knife edge was inserted within the openings of a cutter, sited slightly around the hypothalamus, segmenting the brain into three separate slices. These slices were moved to a glass plate, latent on ice, and dampened with a cold saline solution. The central slice from the dissection was employed to bilaterally isolate the hippocampus, using a sharp scalpel blade. The hippocampus was isolated and preserved at −40 °C for subsequent assessments related to biochemical (right and left hemispheres of the hippocampus were used), neurochemical, and histopathological studies.

### 2.4. Behaviors evaluation

#### 2.4.1. Open-field-test (OFT)

Locomotor activity was evaluated in an open area of specific dimensions 80 cm × 80 cm × 42 cm (*L* × *W* × *H*). OFT is employed to examine locomotion in a novel environment in rodents. An isolated soundproof and well-lit room was used for the activity. The rat was positioned in the center of the apparatus (which was new for rat), and the number of crossings was monitored for 5 minutes.^45^

#### 2.4.2. Home-cage-activity (HCA)

A clear box with specified dimensions 25 cm × 25 cm × 20 cm (*L* × *W* × *H*) was used to evaluate locomotor activity in an environment that was familiar to the rodent.^45^ A home cage with a familiar environment (saw dust-covered floor) was used, and cage crossings (passages and falling/rearing) were monitored for 5 minutes.

#### 2.4.3. Elevated-plus-maze (EPM)

The activity can be utilized to assess anxiety and locomotor activity in a novel environment^47^ The apparatus included 4 arms of which, 2 were closed and 2 were opened. The arms have a similar 50 cm length and 10 cm width. A mid area that connected arms. The apparatus was elevated from the floor at 60 cm. The open arms and the elevation, induce anxiety responses in rodents when placed on the elevated plus maze. Following the treatment, each rat is individually placed in the mid area of the apparatus. Entries in the closed arm and the time passed in the open arm were monitored manually over a 5-minutes duration.

#### 2.4.4. Light-dark-activity (LDA)

The activity was performed to evaluate anxiety-like behaviors.^48^ It involves an enclosure divided by a partition into 2 boxes: a dark box and a light box. The dimensions of these boxes were 26 cm × 26 cm × 26 cm, with a connecting space of 12 cm × 12 cm between them. The light-box is transparent, whereas the dark box is translucent. At the start, rats were positioned in the light-box. Entries in the dark-box and time passed in the light-box were observed manually for 5 minutes.

### 2.5. Biochemical evaluation

Malondialdehyde (MDA) is a residual part of lipid peroxidation; elevated contents of MDA cause oxidative stress. To evaluate the oxidative-stress contents of MDA, and activity of various antioxidant enzymes was measured. The levels of inflammatory cytokines were estimated to evaluate neuroinflammation.

#### 2.5.1. Malondialdehyde (MDA)

The quantification of MDA levels followed a methodology similar to that previously documented.^46^ 0.03 mL of the homogenate of the hippocampus was mixed with 2000 µl of a solution comprising 15% TCA and 0.375% TBA. An ice-cold water bath at 4 °C was used to rapidly cool the mixture after heating it for 20 minutes in a water bath. Afterward, centrifugation was done at 35000 rpm for 10 minutes. An absorbance of 532 nm was measured in the resulting supernatant, which displayed a light pink color. The assessment of lipid-peroxidation was articulated in nmol TBARS per mg of protein.

#### 2.5.2. Superoxide dismutase (SOD)

The SOD activity was assessed by following the procedure previously described.^48^ A 10% homogenized hippocampal tissue sample was treated with ethanol (0.75 mL) and cold chloroform (0.15 mL), and then centrifuged the mixture. The resulting clear liquor was treated with EDTA (0.5 mL, 0.6 mM) and Carbonate-Bicarbonate buffer (1.0 mL, 0.1 M; pH 10.2), epinephrine (0.5 mL, 1.8 mM) was used to start the reaction. The absorbance was checked at 480 nm for three minutes. All reagents except the supernatant were present in the blank test tube.

#### 2.5.3. Catalase (CAT)

The CAT activity was assessed by a previously reported method.^46^ Phosphate buffer (0.1 M, pH 7.0) was used to prepare tissue homogenate. CAT activity was assessed using a reaction mixture consisting of tissue homogenate (0.1 mL) and phosphate buffer (1 mL, pH 7.4). Each tube was then treated with H_2_O_2_ (0.4 mL). Incubation at 37 °C for 90 seconds was conducted on the mixtures. All test tubes appeared blue; after that, potassium-dichromate/acetic acid (2 mL) was mixed to stop the reaction. As a result of the synthesis of chromic acetate, the appearance of the mixtures changed from blue to green after incubation at 100 °C for 15 minutes. There was no tissue homogenate present in the control test tube. Contrary distilled water was used instead of tissue homogenate in the blank test tube. The 570 nm was used to measure the absorbance. The activity of CAT was measured in µmol of H_2_O_2_ consumed/minute per mg of protein in the tissue samples.

#### 2.5.4. Glutathione peroxidase (GPx)

The activity of GPx was determined by an earlier method.^49^ In which sodium azide (0.1 mL) was combined with 0.2 mL of supernatant from brain tissue, glutathione 0.2 mL, and phosphate buffer (0.3 mL) in each test tube and then incubated at 37 °C for 15 minutes. All test tubes containing tissue samples were treated with TCA to arrest the reaction. The supernatant obtained after centrifugation was utilized for subsequent analysis after being centrifuged at 15000 rpm for 5 minutes. A mixture of bis-sodium hydrogen phosphate buffer (0.2 mL), DTNB (0.7 mL), and supernatants from each tissue sample (0.1 mL) was added to each tube. The absorbance was checked at 420 nm. The GPx activity was quantified and expressed as µmol of residual GSH per mg of protein, in line with the provided methodology.

#### 2.5.5. Inflammatory markers

The quantitative analysis of hippocampal IL-6 and TNF-α was performed by the ELISA technique using Abcam's kit.^48^

### 2.6. Neurochemical evaluation

#### 2.6.1. Determination of 5-HT and 5-HIAA

The Hippocampal 5-HT and 5-HIAA were estimated by using previous methods.^50^ The reverse-phase-high-performance-liquid chromatography through an electro-chemical (EC) detector was used for the quantitative analysis of 5-HT and 5-HIAA.

#### 2.6.2. 5-HT1A receptor expression

The Real-Time Polymerase–Chain Reaction (qRT-PCR) was used to evaluate expression of 5-HT1A receptor.^8^

#### 2.6.3. RNA extraction and reverse transcription

Whole RNA was mined from the samples using TRIzol® reagent. For quantitative analysis of the entire RNA, NanoDrop was used. RNA specimens were processed and frozen at a very low temperature (−80 °C) until reverse transcription was executed. Using the Revert-aid-first-strand cDNA synthesis kit and Master-cycler-proS (according to the producer's guidelines), cDNA was produced from RNA (each sample). The synthesized cDNA was then frozen at a low temperature (−20 °C) for further use in qRT-PCR.^50,51^

#### 2.6.4. Quantitative real-time polymerase chain reaction (qRT-PCR)

Table 1 shows the primers for 5-HT1A receptor and β-actin genes' designed by NCBI-Primer BLAST. Using the producer's guidelines RT-PCR was performed with Maxima Green/ROX qPCR Master Mix (2×). A thermal cycling procedure similar to that adopted by Ali *et al.*^50,51^ was used, which consisted of a denaturation stage (at 95 °C, for 10 minutes), subsequently 40 cycles (at 95 °C, for 15 seconds), an annealing stage (at 60 °C for 30 seconds) and an extension stage (at 72 °C for 30 seconds). An HRM software by AriaMx Agilent was used to attain and evaluate the data. An internal control β-actin mRNA was co-amplified with the 5-HT1A receptor mRNA to ensure accuracy. The levels of 5-HT1A receptor mRNA were then normalized to beta-actin mRNA.

**Table 1:** Primer sequence, annealing temperature and amplicon size

Gene	Primers	Annealing temperature (^°^C)	Amplicon size (bp)
β-actin	F: 5′-ACCCACACTGTGCCCATCTA	58.5	285
	R: 5′-CGGAACCGCTCATTGCC	57.1	
5-HT1A receptor	F: 5′-CCCCCCAAGAAGAGCCTGAA	59.4	335
	R: 5′-GGCAGCCAGCAGAGGATGAA	60.1	

### 2.7. Histopathological evaluation

Hematoxylin and Eosin staining were used for histopathological analysis of hippocampal tissue of rat's brain. The earlier reported procedure was used.^44^ The light microscope was set at 400× magnification to visualize and analyze tissue images.

### 2.8. Histopathological analysis

Hippocampal tissue (ventral and dorsal sides) was histopathologically analyzed by hematoxylin and eosin staining.^48^ The previously described process was applied. For the purpose of viewing and analyzing tissue pictures, the light microscope was adjusted to 400X magnification.

### 2.9. Statistical analysis

To assess the effects of GAL and OMP by using SPSS Ver. 20.0 two-way Anova followed by ‘Tukey's test’ was used to assess all the data. GAL and OMP were used as independent variables. *p* ˂ 0.05 was deliberated as substantial.

## 3. Results

### 3.1. Impact of GAL on OMP-induced change in locomotor activity

The locomotor activity in the familiar environment (HCA) and novel environment (OFA) was observed (Fig. 2). Statistical analysis executed by two-way Anova for the results of square crossed in OFA (Fig. 2a) disclosed substantial impacts of GAL [*f*_(2,42)_ = 205.05, *p* = 0.001], OMP [*f*_(1,42)_ = 8.48, *p* = 0.006], and interface of GAL and OMP [*f*_(2,42)_ = 21.09 *p* = 0.001]. Tukey's test exhibits reduced squares crossing in OMP-administered rats than in VEH-treated rats. Both doses of GAL increased squares crossing in VEH and OMP-administered animals than VEH + VEH and OMP-administered groups.

**Fig. 1:**
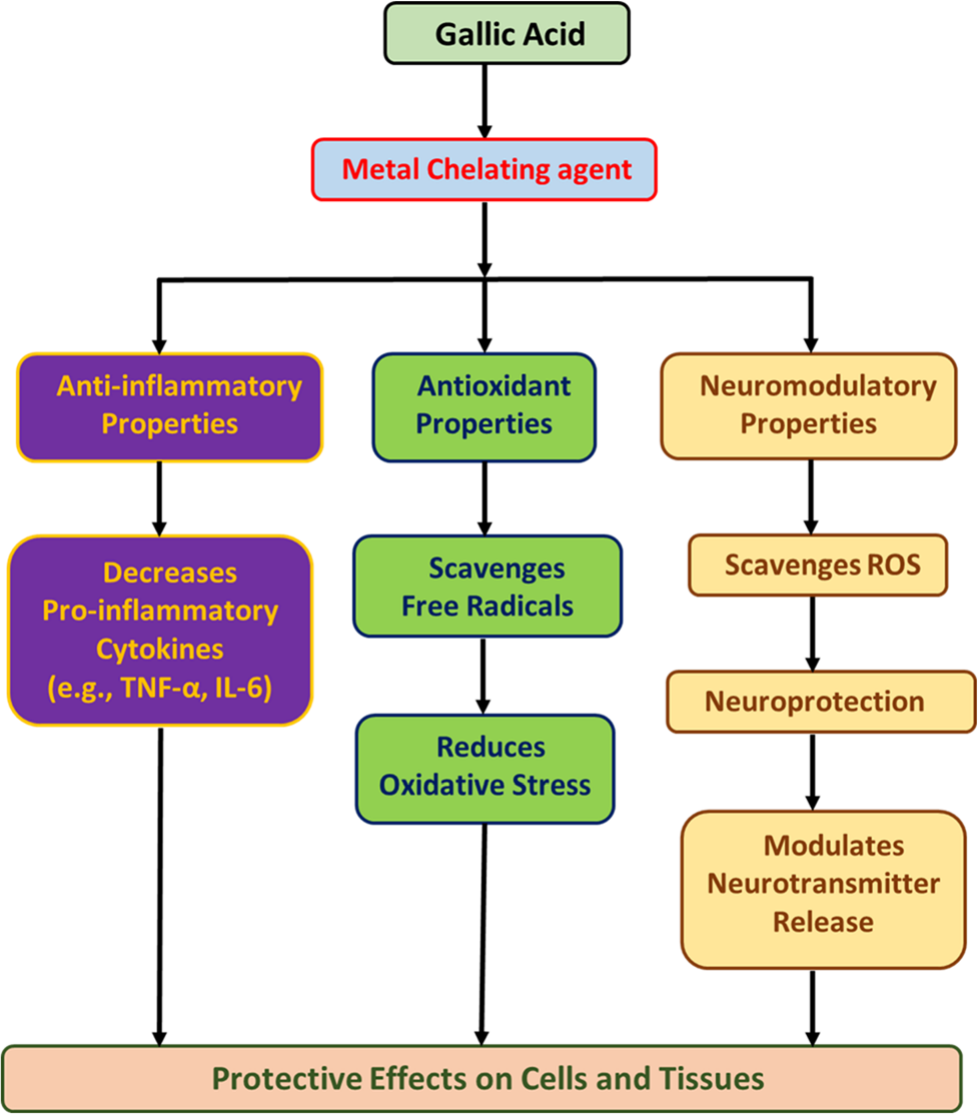
A schematic diagram summarizing the mechanism of gallic acid.

**Fig. 2:**
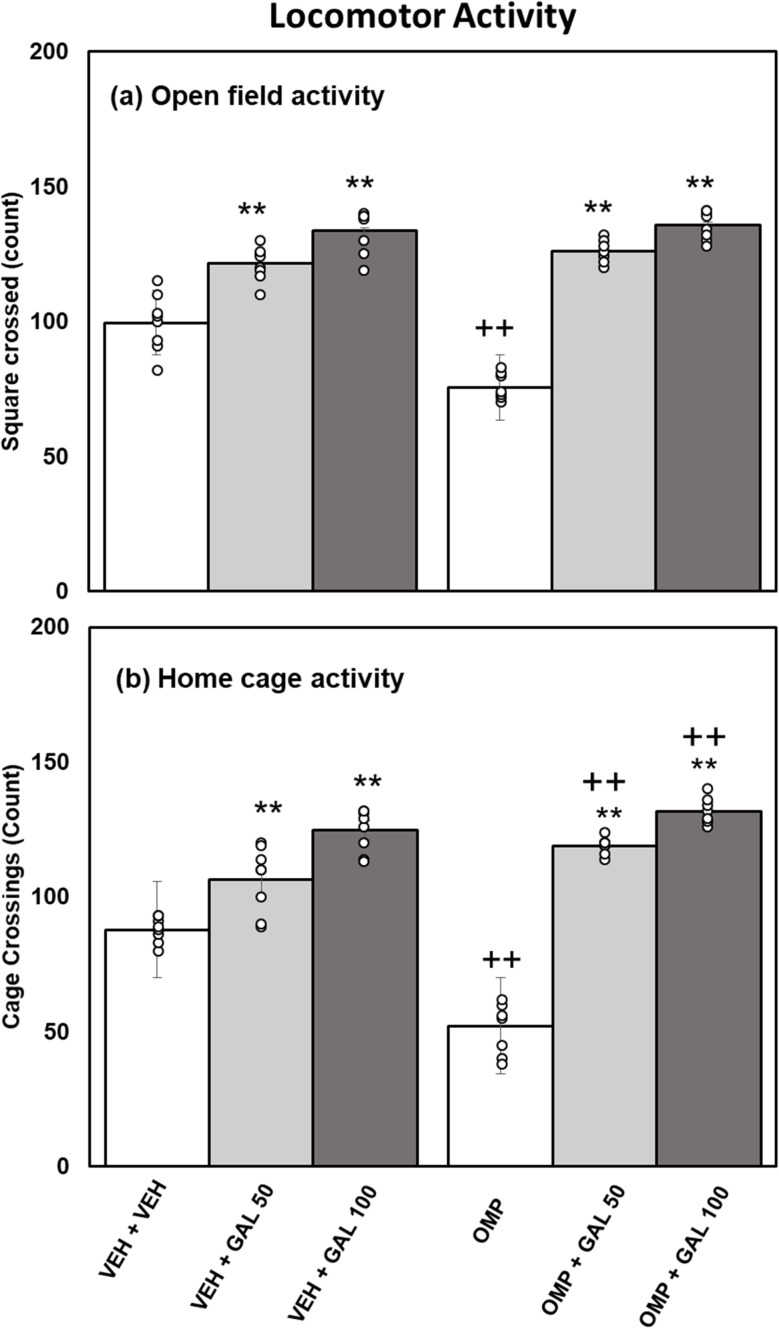
Effect of GAL on locomotor activity (a) open field activity (b) home cage activity in VEH and OMP treated animals [mean ± SD (*n* = 8)]. Data was analyzed by two-way ANOVA and followed by Turkey's test showed a significant difference as **p < 0.01 when compared with VEH + VEH and OMP, ++*p* < 0.01 when compared with VEH + VEH, VEH + GAL 50, VEH + GAL 100 treated animals.

Statistical analysis executed by two-way Anova for the results of cage crossings in the HCA (Fig. 2b) disclosed substantial impacts of GAL [*f*_(2,42)_ = 242.51, *p* = 0.001], OMP [*f*_(1,42)_ = 5.88, *p* = 0.02], and interface of GAL and OMP [*f*_(2,42)_ = 46.63 *p* = 0.002]. Tukey's test exhibits cage crossings reduced in OMP-administered rats than in VEH-treated rats. Both doses of GAL increased cage crossings in VEH and OMP-administered animals than the VEH + VEH and OMP-treated groups. The cage crossings were higher in counts in OMP + GAL (both doses) than in VEH + GAL (both doses) treated animals.

### 3.2. Impact of GAL on OMP-induced anxiety-like behavior in LDA

**Fig. 3:**
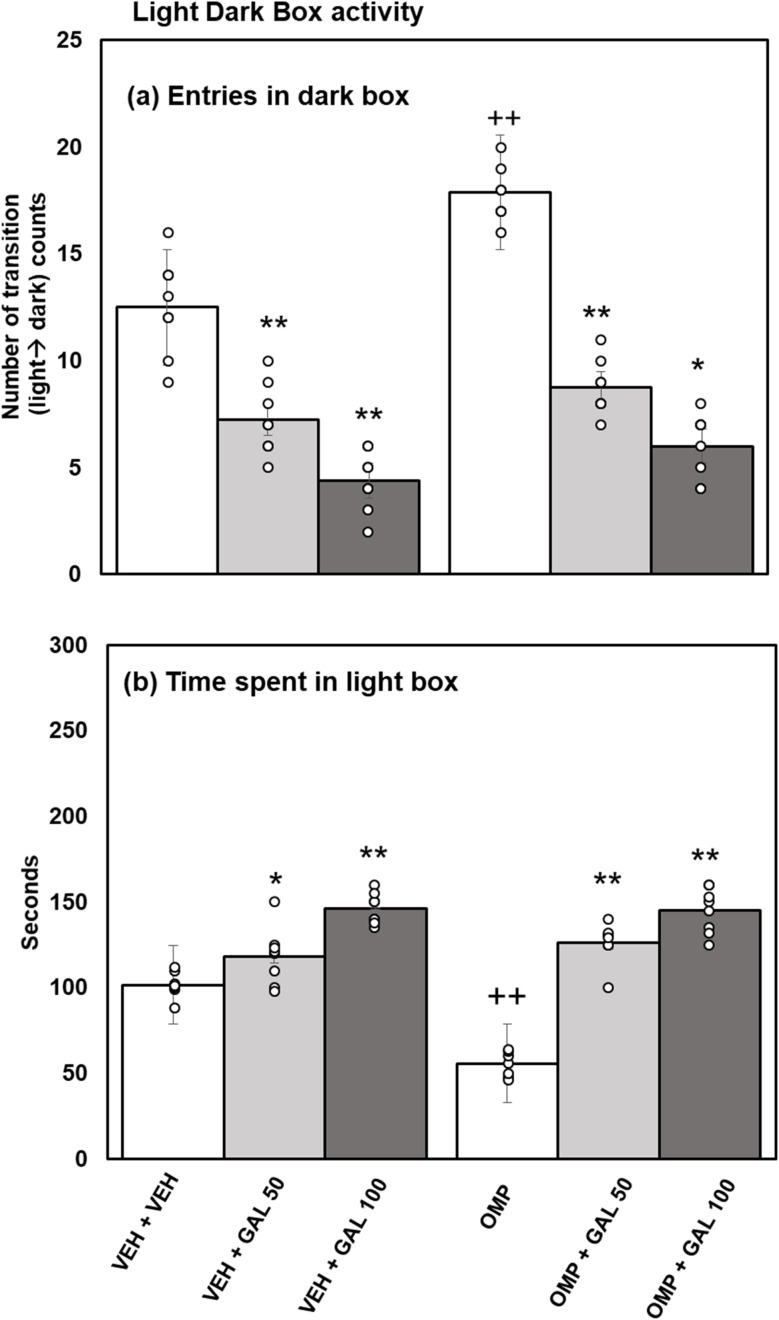
Effect of GAL on anixety profile in VEH and OMP treated animals evaluated *via* light dark activity box (a) entries in dark box (b) time spent in light box [mean ± SD (*n* = 8)]. Data was analyzed by two-way ANOVA and followed by Turkey's test showed a significant difference as **p < 0.01 *p < 0.05 when compared with VEH + VEH and OMP, ++*p* < 0.01 when compared with VEH + VEH treated animals.

The time expended in the light-box and entries in the dark-box of LDA were recorded as a measure of anxiety profile (Fig. 3). Statistical analysis executed by two-way Anova for the results of entries in the dark-box (Fig. 3a) disclosed substantial impacts of GAL [*f*_(2,42)_ = 165.86, *p* = 0.001], OMP [*f*_(1,42)_ = 37.55, *p* = 0.001] and interface of GAL and OMP [*f*_(2,42)_ = 7.56, *p* = 0.002]. Tukey's test exhibits enhanced entries in the dark-box in OMP-administered than in VEH-treated rats. Both doses of GAL reduced entries in the dark-box of VEH and OMP-administered animals than VEH + VEH and VEH + OMP administered groups.

Statistical analysis executed by two-way Anova for the results of time expended in the light-box (Fig. 3b) disclosed substantial impacts of GAL [*f*_(2,42_ = 145.05, *p* = 0.001], OMP [*f*_(1,42_ = 15.86, *p* = 0.001] and interface of GAL and OMP [*f*_(2,42_ = 26.23 *p* = 0.0001]. Tukey's test exhibits, reduced time expended in light-box in OMP-administered than VEH-treated rats. Both doses of GAL notably enhanced the time expended in light-box of VEH and OMP-administered rats than VEH + VEH and OMP-administered groups.

### 3.3. Impact of GAL on OMP-induced anxiety-like behavior in EPM

**Fig. 4:**
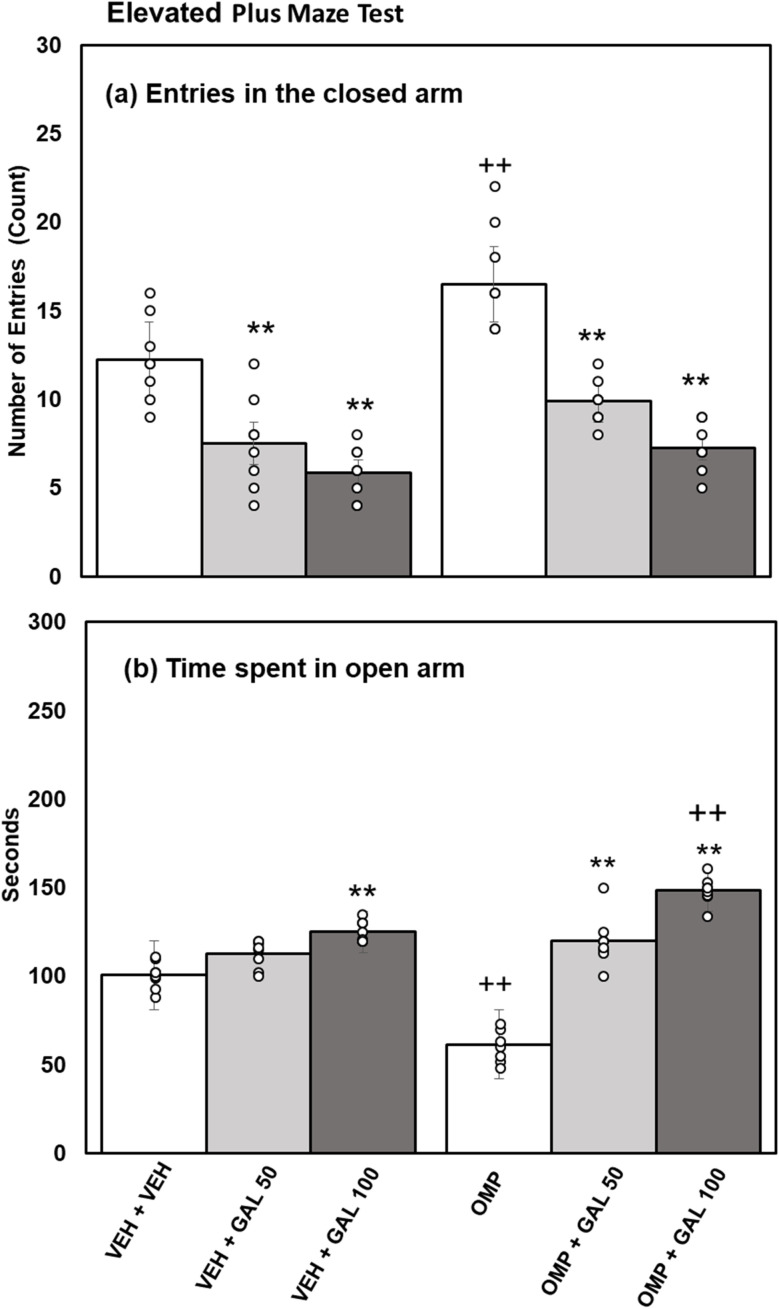
Effect of GAL on anixety profile in VEH and OMP treated animals evaluated *via* plus maze activity box (a) entries in closed arm (b) time spent in open arm [mean ± SD (*n* = 8)]. Data was analyzed by two-way ANOVA and followed by Turkey's test showed a significant difference as **p < 0.01 when compared with VEH + VEH and OMP, ++*p* < 0.01 when compared with VEH + VEH, VEH + GAL 100 treated animals.

The time expended in the open arm and entries in the closed-arm of EPM were recorded as a measure of the anxiety profile (Fig. 4). Statistical analysis executed by two-way Anova for the results of entries in the closed-arm (Fig. 4a) showed significant effects of GAL [*f*_(2,42_ = 53.55, *p* = 0.001] and OMP [*f*_(1,42_ = 17.50, *p* = 0.001]. The interaction of GAL and OMP [*f*_(2,42_ = 1.748, *p* = 0.187] was insignificant. Tukey's test exhibits enhanced entries in the closed-arm in OMP-administered than VEH-treated rats. Both doses of GAL reduced entries in the closed-arm of VEH and OMP-administered animals than VEH + VEH and OMP-treated groups.

Statistical analysis executed by two-way Anova for the results of time expended in the open-arm (Fig. 4b) disclosed substantial impacts of GAL [*f*_(2,42)_ = 149.23, *p* = 0.001], the non-substantial impact of OMP [*f*_(1,42)_ = 1.17, *p* = 0.286]. The interface of GAL and OMP [*f*_(2,42)_ = 49.19, *p* = 0.001] was also substantial. Tukey's test exhibits reduced time expended in the open-arm in OMP-administered rats than VEH-treated rats. Both doses of GAL enhanced the time expended in the open-arm of VEH and OMP-administered animals than VEH + VEH and OMP-treated groups.

### 3.4. Impact of GAL on neuroinflammation induced by OMP

**Fig. 5:**
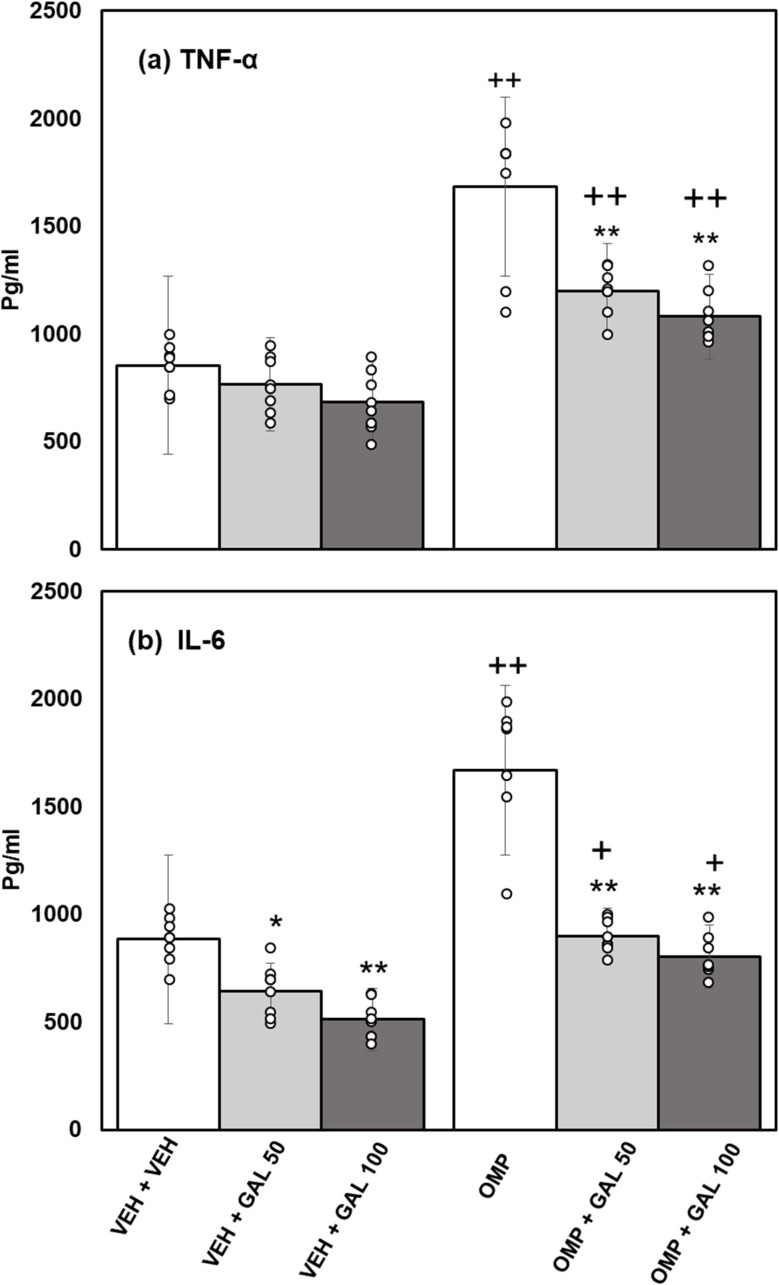
Effect of GAL on hippocampal inflammatory markers (a) TNF-∝ (b) IL-6 in VEH and OMP treated animals [mean ± SD (*n* = 8)]. Data was analyzed by two-way ANOVA and followed by Turkey's test showed a significant difference as **p < 0.01 when compared with VEH + VEH and OMP, ++*p* < 0.01 when compared with VEH + VEH, VEH + GAL 50, VEH + GAL 100 treated animals.

The impacts of GAL on hippocampal inflammatory markers followed by OMP administration in rats are shown in Fig. 5. Data analysis for TNF-α (Fig. 5a) was assessed by two-way Anova which displayed a substantial effect of GAL [*f*_(2,42)_ = 20.62, *p* = 0.001], OMP [*f*_(1,42)_ = 117.36, *p* = 0.001], and the interface of GAL and OMP [*f*_(2,42)_ = 7.32, *p* = 0.002]. Tukey's test exhibits increased contents of TNF-α in the brain tissue of OMP-treated rats. The administration of GAL (at both doses) decreases TNF-α levels in OMP-administered rats. The contents of TNF-α were greater in OMP + GAL (both doses) than in VEH + GAL treated rats.

Data analysis for IL-6 (Fig. 5b) was evaluated by two-way Anova, which displayed a substantial effect of GAL [*f*_(2,42)_ = 75.94, *p* = 0.001], OMP [*f*_(1,42)_ = 103.76, *p* = 0.001], and the interaction of GAL and OMP [*f*_(2,42)_ = 15.32, *p* = 0.001]. Tukey's test exhibits increased contents of IL-6 in the hippocampus of OMP-treated rats. GAL (at both doses) diminished IL-6 contents in VEH and OMP-treated rats. The content of IL-6 was higher in OMP + GAL (both doses) than in VEH + GAL (both doses) treated rats.

### 3.5. Impact of GAL on the oxidative stress and altered activity of antioxidant enzyme induced by OMP

**Fig. 6:**
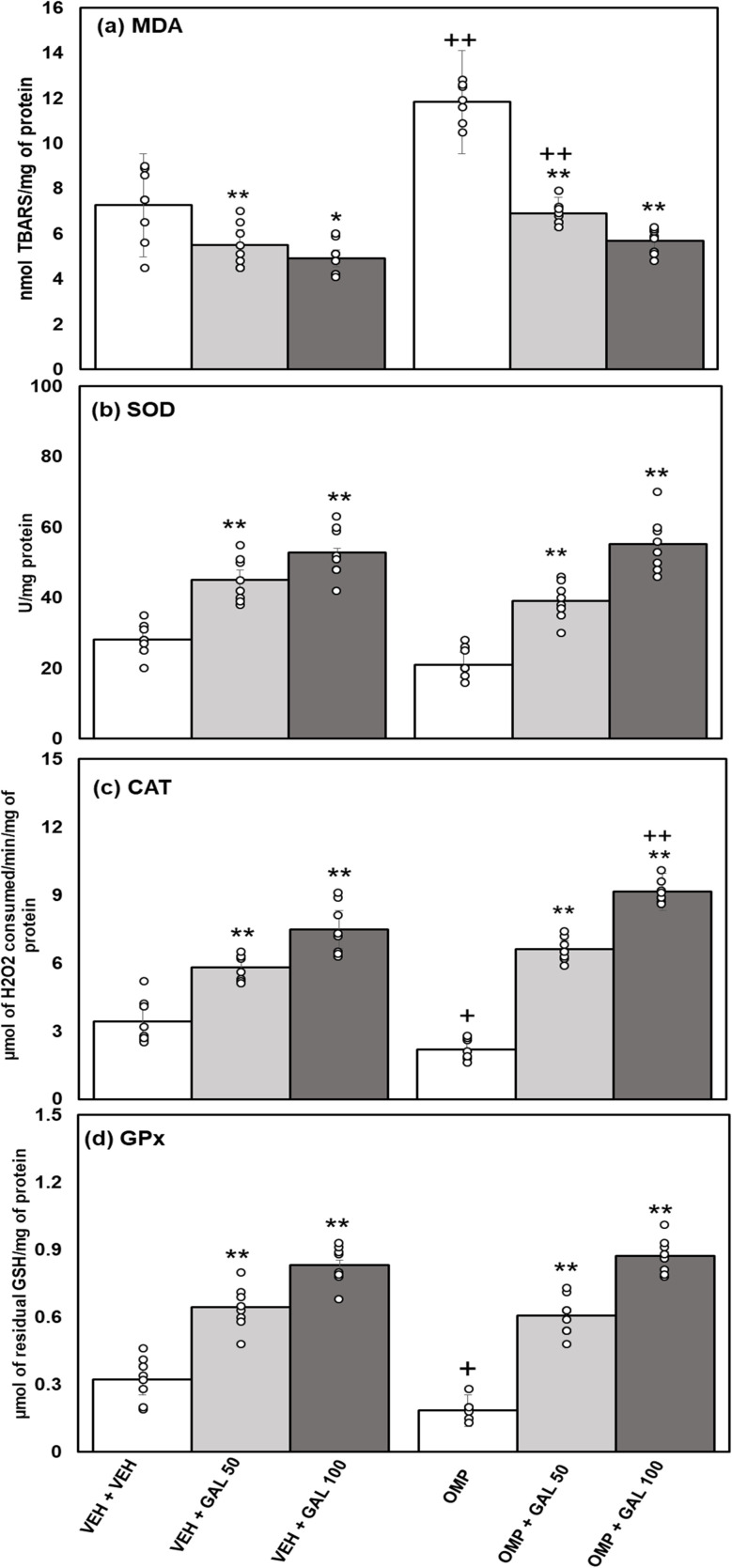
Effect of GAL on hippocampal of oxidative stress markers (a) MDA and activity of antioxidant enzymes (b) SOD (c) CAT (d) GPx in VEH and OMP treated animals [mean ± SD (*n* = 8)]. Data was analyzed by two-way ANOVA and followed by Turkey's test showed a significant difference as **p < 0.01 when compared with VEH + VEH and OMP, ++*p* < 0.01 +*p* < 0.05 when compared with VEH + VEH, VEH + GAL 50, VEH + GAL 100 treated animals.

The impact of GAL on hippocampal MDA levels and the activity of antioxidant enzymes followed by OMP administration in rats, is shown in Fig. 6. Data on MDA (Fig. 6a) statistically analyzed by two-way Anova displayed substantial impacts of GAL [*f*_(2,42)_ = 82.73, *p* = 0.001], OMP [*f*_(1,42)_ = 65.28, *p* = 0.001], and the interface of GAL and OMP [*f*_(2,42)_ = 16.91, *p* = 0.001]. Tukey's test exhibits enhanced contents of MDA in the hippocampus of OMP-treated rats. The administration of GAL (at both doses) decreases MDA contents in VEH and OMP-administered rats. OMP + GAL 50 administered animals exhibited increased MDA than VEH + GAL treated rats.

Analysis of data on SOD activity (Fig. 6b) was assessed by two-way Anova which displayed a substantial impact of GAL [*f*_(2,42)_ = 93.62, *p* = 0.001], OMP [*f*_(1,42)_ = 4.11, *p* = 0.049], and the interface of GAL and OMP [*f*_(2,42)_ = 2.89, *p* = 0.06]. Tukey's test shows comparably reduced SOD activity in the brain region of OMP-treated rats. GAL (at both doses) enhanced SOD activity in VEH and OMP-treated rats.

Analysis of data on CAT activity (Fig. 6c) was assessed by two-way Anova, which displayed a substantial impact of GAL [*f*_(2,42)_ = 229.40, *p* = 0.001], OMP [*f*_(1,42)_ = 3.715, *p* = 0.061], and the interface of GAL and OMP [*f*_(2,42)_ = 16.28, *p* = 0.001]. Tukey's test exhibits reduced activity of CAT in the hippocampus of OMP-treated rats. GAL (at both doses) enhanced the activity of CAT in VEH and OMP-treated rats. The activity of CAT was greater in OMP + GAL 100 than in VEH + GAL 100-treated rats.

Analysis of data on GPx activity (Fig. 6d) was assessed by two-way Anova which displayed a substantial impact of GAL [*f*_(2,42)_ = 213.03, *p* = 0.001], OMP [*f*_(1,42)_ = 3.03, *p* = 0.089], and the interaction of GAL and OMP [*f*_(2,42)_ = 5.373, *p* = 0.008]. Tukey's test exhibits reduced activity of GPx in the brain tissue of OMP-treated rats. GAL boosted GPx activity in VEH and OMP-treated rats.

### 3.6. Impact of GAL on OMP-induced alterations in the concentrations of 5-HT and its metabolite and 5-HT1A receptor expression levels

**Fig. 7:**
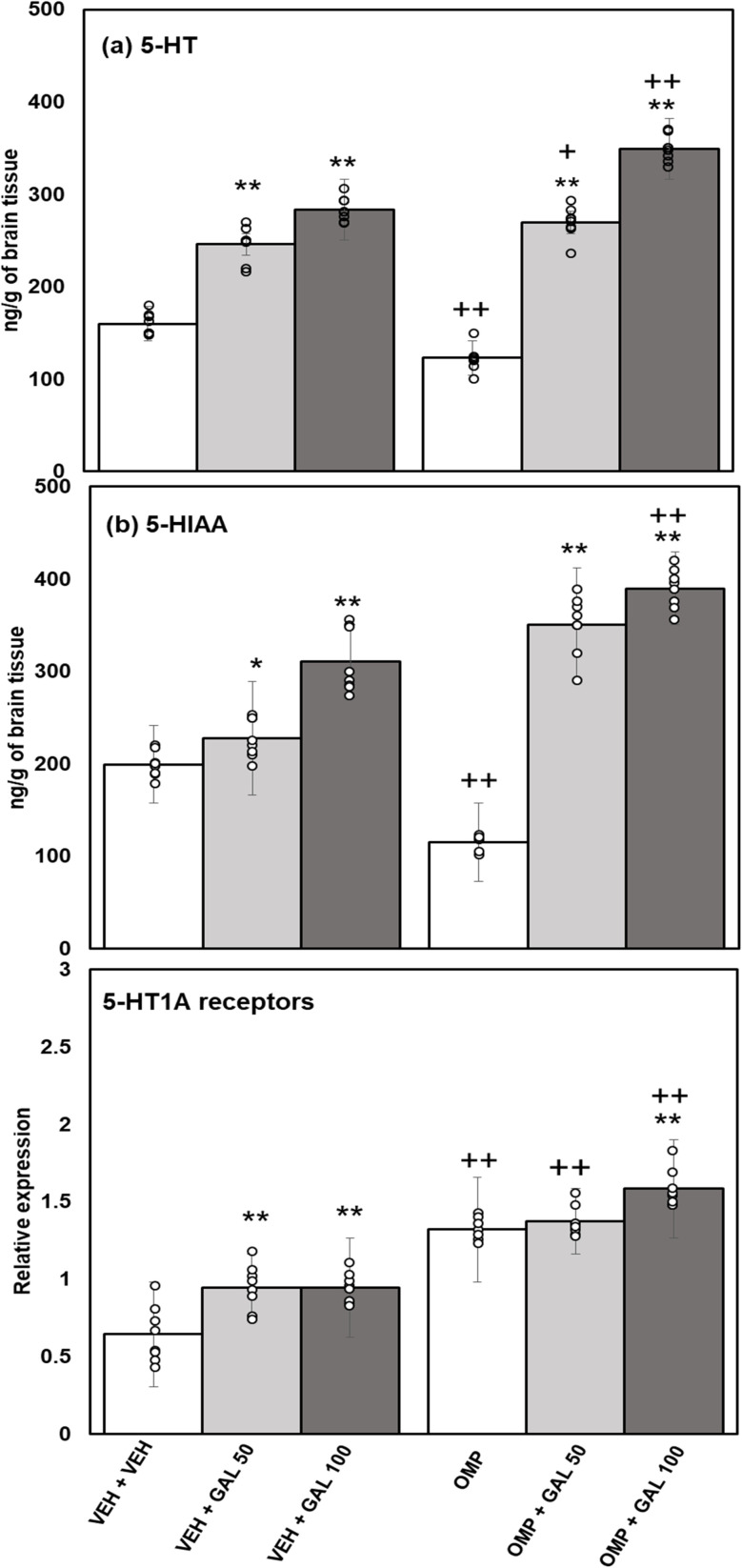
Effect of GAL on hippocampal of oxidative stress markers (a) 5-HT (b) 5-HIAA receptor expression levels in VEH and OMP treated animals [mean ± SD (*n* = 8)]. Data was analyzed by two-way ANOVA and followed by Turkey's test showed a significant difference as **p < 0.01 *p < 0.05 when compared with VEH + VEH and OMP, ++*p* < 0.01 +*p* < 0.05 when compared with VEH + VEH, VEH + GAL 50, VEH + GAL 100 treated animals.

The impact of GAL on hippocampal 5-HT, 5-HIAA, and 5-HT1A receptor expression levels following OMP administration in rats is shown in Fig. 7. Data analysis for 5-HT (Fig. 7a) was assessed by two-way Anova, which displayed a substantial effect of GAL [*f*_(2,42)_ = 566.06, *p* = 0.001], OMP [*f*_(1,42)_ = 16.52, *p* = 0.001], and the interface of GAL and OMP [*f*_(2,42)_ = 47.57, *p* = 0.001]. Tukey's test exhibits that 5-HT contents, reduced in OMP-treated rats. The administration of GAL (at both doses) elevated 5-HT contents in VEH and OMP-treated rats. The contents of 5-HT were greater in OMP + GAL than in VEH + GAL. The levels of 5-HT were greater in OMP + GAL 50 and 100 treated rats than in VEH + GAL 50 and 100 treated rats.

Data analysis for 5-HIAA (Fig. 7b) was assessed by two-way Anova, which displayed a significant effect of GAL [*f*_(2,42)_ = 276.07, *p* = 0.001], OMP [*f*_(1,42)_ = 32.83, *p* = 0.001], and the interaction of GAL and OMP [*f*_(2,42)_ = 84.77, *p* = 0.001]. Tukey's test exhibits decreased 5-HIAA contents in OMP-treated rats. The administration of GAL (at both doses) increased 5-HIAA contents in VEH and OMP-administered rats. The contents of 5-HIAA were greater in OMP + GAL 100 treated than in VEH + GAL 100-treated rats.

Data analysis for 5-HT1A receptor expression levels (Fig. 7c) assessed by two-way Anova displayed a substantial effect of GAL [*f*_(2,42)_ = 20.97, *p* = 0.001], OMP [*f*_(1,42)_ = 265.08, *p* = 0.001], and the interface between GAL and OMP [*f*_(2,42)_ = 3.486, *p* = 0.001]. Tukey's test shows reduced expression levels of 5-HT-1A receptors in OMP-treated rats. The administration of GAL (at both doses) enhanced the expression of 5-HT1A receptors in VEH and OMP-administered rats. The 5-HT1A receptor expression was higher in OMP + GAL than in VEH + GAL-treated rats.

### 3.7. Impact of GAL on histopathological changes in the hippocampus induced by OMP

**Fig. 8:**
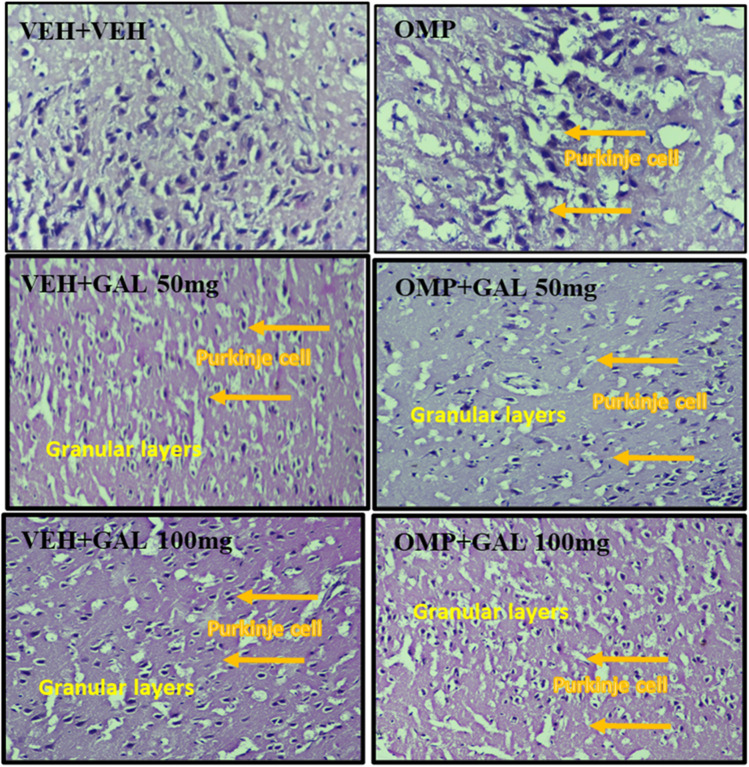
Microscopic representing histopathological alterations using hematoxylin and eosin staining at 400X in the rat brain [hippocampus dosal and ventral part)] of VEH + VEH, VEH + GAL (50 mg kg^−1^), VEH + GAL (100 mg kg^−1^) OMP, OMP + GAL (100 mg kg^−1^) treated animals.

The histopathology changes in hippocampal tissues of VEH + VEH, VEH + GAL 50 mg, VEH + GAL 100 mg, OMP, OMP + GAL 50 mg, and OMP + GAL 100 mg are shown in Fig. 8. The micrograph of VEH + VEH showed, that increased bare places and pyknotic nuclei in this group are signs of perivascular rapping, as it may be visible in the vicinity of areas and undergoing degenerative changes. Some areas show signs of nuclear disintegration. As a result, the data demonstrate that the cells are neatly organized and their morphology is effectively preserved. The micrograph of VEH + GAL 50 mg and VEH + GAL 100 mg showed fewer vacant areas and fewer degenerative changes, indicating that their core structure is effectively maintained. Satellite cells, on the other hand, were dispersed and in greater numbers. Perivascular cuffing can be noticed in the region of the neuronal cells' darkly pigmented pyknotic nucleus in a few spots. As a result, these groups' morphology is normalized. The micrograph of OMP administered rats showed the presence of vacuolated cytoplasm in pyramidal neurons. The chromatin was marginated to one side in a few locations, and the nuclei had a vesicular appearance. There are foamy aggregates in some regions that are free of pyramidal neuronal structures. As a result, OMP treatment induced neurodegeneration and pathological abnormalities in cells. The micrograph of OMP + GAL 50 mg and OMP + GAL 100 mg showed pyramidal neuronal cells were found to be shrunken, with hyperchromatic morphology and vacuolated cytoplasm. Some pyramidal neuronal cells contain pyknotic nuclei, whereas others have vesicular appearances. However, glial cells can be detected and distributed around some neural processes. Overall results showed minor pathological changes.

## 4. Discussion

The current work for the first time reports the effect of various doses of GAL (50 and 100 mg kg^−1^) on OMP-induced anxiety-like behavior, hypoactivity, oxido-neuroinflammation, altered serotonin mechanism, and histopathology of the hippocampus in rats. Previous study showed that OMP at the dose of 20 mg kg^−1^, already used in clinical settings, decreased exploratory/locomotor activity (Fig. 2) and induced anxiety-like symptoms (Fig. 3 and 4) in various behavioral analysis *i.e.* OFA, HCA, LDA, EPM). The administration of OMP altered 5-HT metabolism and 5-HT1A receptor relative expression (Fig. 7), increased inflammatory markers (Fig. 5), and oxidative stress (Fig. 6) with a decreased defensive antioxidant enzyme (Fig. 6) mechanism. The morphology of the hippocampus (Fig. 8) also deteriorated following the treatment of OMP. Conversely, various doses of GAL prevented the OMP-induced adverse effect, produced the anxiolytic effect, and improved locomotor activity. It is observed that GAL produced a protective effect (Fig. 1).

OMP, a known PPI, is widely used to treat hyperacidity and gastric reflux.^52^ In an earlier study, an OFT was used to evaluate motor alteration following memory performance in an animal model.^53^ The OFT and HCA were used to evaluate locomotion activity in the present research work. The finding of the present study showed that OMP intake decreased squares crossed in open field activity (novel environment) and cage crossings in home cage activity (familiar environment) (Fig. 2a and b), which proved earlier^8^ and showed that OMP decreased locomotor activity. It is also well documented that IP injection can also induce physiological stress responses and behavioral changes *i.e.*, increased anxiety-like behavior^54^ in experimental rodents. In the present study, we can avoid the stress induced by IP injection because all animals received the same treatment. LDA and EPM were used as a pharmacologically approved model to evaluate anxiety profiles in experimental animals.^40,50^ Fear/anxiety conditions disturb the animals' locomotor activity during LDA and EPM tests, and animals are inclined to stay in the dark box and closed arms respectively.^55^ It is supposed that drug-induced increases in open-arm exploration, without associated increases in locomotor activity, reflect an anxiolytic effect.^56^ OMP increased entries in the dark box and reduced time spent in the light box of LDA (Fig. 3a and b) and increased entries in the closed arm and decreased time spent in the open arm of EPM (Fig. 4a and b), suggesting the anxiogenic effect of OMP as reported previously.^51^ A noticeable reduction in exploratory/locomotor activity in OFA and HCA was witnessed in the present study for OMP-administered animals, supporting the view that OMP is an anxiogenic drug. The LDA and EPM behavioral tests confirm our results from OFA and HCA as time spent in the light box and open arm decreased and entries in the dark box and closed arm increased respectively in OMP-administered rats. Previously, PPIs intake with increased blood gastrin levels had been associated with anxiety in clinical studies^57^ because cholecystokinin type B receptor (CCKB) stimulation was observed in the brain that led to decreased locomotor activity and anxiety-like behavior.^58^ In the present study, OMP-induced decreased behavioral activities may be linked with reduced 5-HT metabolism (Fig. 8), increased oxido-neuroinflammation (Fig. 5 and 6), and reduced antioxidant enzymes (Fig. 7) which is in agreement with the previous report.^8,59^

Enhanced oxidative stress leads to neurotoxicity and plays an important role in psychiatric illness.^60^ Extensive published data showed that OMP increased oxidative stress^59,61^ with increased biomarkers *i.e.*, MDA, reduced antioxidant enzymes (SOD, CAT, GPx) activity that led to increased levels of inflammatory markers.^62^ SOD as an antioxidant enzyme initiates the defensive mechanism by neutralizing superoxide radicals into hydrogen peroxide, concurrently, CAT and GPx change hydrogen peroxide into water and molecular oxygen.^48^ The present study shows that OMP can increase MDA levels (Fig. 5) and reduce the activity of antioxidant enzymes (Fig. 7). The increased oxidative stress can trigger the release of inflammatory cytokines. Previously, OMP administration increased the production of free radicals, MDA, and IL-6 and TNF-α.^59^ Few previous studies^63,64^ showed the upregulation of SOD and CAT by short-term intake of OMP with decreased MDA levels in mice. The outcomes received in those studies for OMP treatment and oxidative stress parameters are not relatable to the present study. The results of the present study agree with the previous reports^59,61^ and indicate that OMP causes neuroinflammation and oxidative deterioration involving anxiety and hypoactivity. Studies have shown that GAL increases exploratory activity in OFA^65^ and prevents locomotion dysfunction^66^ The present finding exhibits that GAL (50 and 100 mg kg^−1^) increased locomotion in OFA and HCA (Fig. 2) alone and in OMP-treated rats as reported earlier,^67^ due to its antioxidant, anti-inflammatory, and neuromodulatory potential. Simultaneously, time spent in the light box and open arm was increased and entries in the dark box and closed arm were decreased in LDA and EPM by GAL (Fig. 3 and 4) alone and in OMP-administrated rats. Earlier studies reported that various doses of GAL *i.e.*, 20 and 30 mg kg^−1^,^68^ 50 and 100 mg kg^−1^,^40,69^ and, 50, 100, and 150 mg kg^−1^^70^ due to antioxidant and anti-inflammatory potential produced anxiolytic-like effects and increased locomotion. Conversely, high doses of GAL (500 mg kg^−1^) produced sedative effects.^24^ In the present study, GAL recovers OMP-induced behavioral deficits by increasing the enzymatic antioxidant system (Fig. 6), inhibiting oxidative deterioration (Fig. 6), and inhibiting neuroinflammatory cytokines (Fig. 5) in rats. It is suggested that OMP-instigated oxidative damages, neuroinflammation, hypolocomotion, and anxiety-like symptoms were vetoed by GAL at both doses, possibly through its antioxidant and neuromodulatory properties.

5-HT is involved in various psychiatric illnesses, such as anxiety, in experimental and clinical studies.^71,72^ Oxidative stress increases 5-HT release^45^ and metabolism in the brain of rodents^22^ and in numerous brain regions (*i.e.*, hippocampus;.^73^ In the present study, repeated administration of OMP decreased 5-HT and 5-HIAA levels (Fig. 7), increased oxido-neuroinflammation in the hippocampus (Fig. 5 and 6), and produced hypolocomotion (Fig. 2) and anxiety-like behavior (Fig. 3 and 4). Previous studies showed that GAL increased 5-HT metabolism in many experimental studies.^69,74^ In the present study, OMP-induced reduction in 5-HT metabolism (Fig. 7) is regulated by both doses of GAL treatment. The findings can be associated with the relative receptor expression of 5-HT1A (Fig. 7). It was also observed that OMP increased 5-HT1A receptor expression (Fig. 7) and decreased 5-HT metabolism (Fig. 7), however, GAL decreased 5-HT1A receptor expression and regulated/normalized 5-HT metabolism and behavioral deficits alone and in OMP-treated animals. Other influences *i.e.*, oxido-neuroinflammation, may be elaborated in the OMP-persuaded effect on 5-HT release and 5-HT metabolism by deteriorating 5-HT neurons. In contrast, GAL, due to its powerful antioxidant potential and neuro-modulatory effects, controlled the oxido-neuroinflammation and inhibited the OMP-induced behavioral and neurochemical deficits.

Photomicrographs (Fig. 8) showed that GAL at both doses resumed the hippocampus morphology following OMP administration. Results showed that oxidative stress darkly stained the cells in the OMP group, which indicates cellular oxidative deterioration.^45^ A noticeable vacuolization and mass of pyramidal neurons following OMP intake revealed neuronal degeneration. On the contrary, GAL administration (both doses) in VEH and OMP-treated rats showed normal and organized cell arrangement. Nevertheless, GAL at both doses averted the behavioral and neurochemical deficits and morphological changes induced by OMP.

This work serves as a foundational study, paving the way for future receptor-specific and signaling-pathway analyses. The limitation of the present work, which boosts the study to a more novel investigation strategy such as quantitative analysis of other neurotransmitters involved in anxiety such as adrenaline, noradrenaline, dopamine, *etc.*, relative expression of the serotonergic receptor (5-HT-2C and others), and immunohistochemistry of various regions, would help grasp the GAL mechanism as a neuromodulator agent that reduces OMP (PPI)-induced psychiatric illness. Although this study is not directly provided molecular evidence for 5-HT1A receptor modulation, but in future work this would be insightful. In this study, we used effective doses of GAL, reported previously; here, we recommend that ineffective doses of GAL should be used against OMP-induced adverse effects. In the current work, the treatment period was 4 weeks; in the future, the impact of long-term treatment of OMP and GAL on behavioral and biochemical parameters could be more interesting and insightful. This study's dependence on β-actin as the only housekeeping gene for normalization without first confirming its stability in the experimental setting is one of its limitations. The precision of gene expression analysis may be impacted by variations in reference gene expression. To guarantee accurate normalization, housekeeping gene validation should be a part of future research.

## 5. Conclusion

In conclusion, this study shows that GAL has strong neuroprotective, neuromodulatory, and antioxidant qualities that work together to lessen the behavioral abnormalities brought on by OMP, particularly anxiety and hyperlocomotion. The results imply that GAL may counterbalance the disturbances caused by OMP by normalizing serotonergic pathways, which would mediate its beneficial effects. These findings highlight the potential therapeutic benefits of taking GAL as a supplement or as part of the diet, as it may be used as a supportive intervention to treat anxiety and related neurobehavioral disorders linked to long-term PPI usage. Further clinical research is necessary to validate GAL's efficacy and safety in people, since its protective mechanisms present a viable route for supplementary therapy in light of the growing prevalence of PPI-induced cognitive adverse effects.

## Ethical approval

All animal experimental procedures were reviewed and approved by the Departmental Bioethical Committee (Approval No. D-1893/2021/Biochem; dated 11/04/2021). The study was conducted in full compliance with the ARRIVE 2.0 guidelines and The Guide for the Care and Use of Laboratory Animals (8th Edition, 2011). All efforts were made to minimize animal suffering and to ensure the highest standards of animal welfare throughout the experimental procedures.

## Author contributions

N. S: conceptualization, supervision, methodology, writing – original draft, project administration, investigation, N. M.: writing – original draft, methodology, investigation, S. S.: formal analysis, visualization, writing – review & editing., S. K.: formal analysis, visualization, writing – review & editing; U. E.: validation, data curation, resources, M. A.: formal analysis, methodology, writing – review & editing; S. A.; funding acquisition, validation, formal analysis, data curation, B. B. K. A.: resources, writing – review & editing, methodology, data curation, A. I.: visualization, funding acquisition, writing – review & editing, formal analysis, data curation; Y. A. B. J: esources, investigation, funding acquisition, project administration, formal analysis, writing – review & editing.

## Conflicts of interest

The authors declare that they have no known competing financial interests or personal relationships that could have appeared to influence the work reported in this paper.

## Funding

This research is funded by the Ongoing Research Funding Program (ORF-2026-457), King Saud University, Riyadh, Saudi Arabia.

## Data Availability

All data supporting the findings of this study are included within the manuscript. Additional data related to this study are available from the corresponding author or can be obtained upon reasonable request at noreen.samad@bzu.edu.pk.
